# Trends and Outcomes in Acute Colonic Diverticulitis: A Single-Center Three-Year Retrospective Cohort Study From Greece

**DOI:** 10.7759/cureus.100480

**Published:** 2025-12-31

**Authors:** Michail Angelos Papaoikonomou, Europi Michailidou, Aggeliki Chlorou, Dimitrios Apostolou, Nikolaos Krokos

**Affiliations:** 1 Department of General Surgery, Agios Pavlos General Hospital, Thessaloniki, GRC; 2 Department of Radiology, Agios Pavlos General Hospital, Thessaloniki, GRC

**Keywords:** acute colonic diverticulitis, epidemiological trends, gastrointestinal surgery, non-operative treatment, recurrence

## Abstract

Background: Acute colonic diverticulitis (ACD) is an increasingly common condition with shifting epidemiological trends, particularly among younger adults in Western countries. The incidence appears to be rising in Greece; however, regional data are scarce. This three-year monocentric cohort study aims to shed light on the epidemiology and clinical outcomes of ACD in a Greek hospital.

Methods: This retrospective cohort study evaluated 60 patients with computed tomography (CT)-confirmed ACD managed between 2022 and 2024, stratified by age in two groups (<65 vs. ≥65 years). Data encompassed demographics, body mass index (BMI), comorbidities, clinical presentation, Hinchey classification, therapeutic interventions, and recurrence rates, with statistical analyses assessing associations between recurrence and patient characteristics.​

Results: Of the 60 patients, 68.3% were under 65 years, and 81.6% were overweight or obese. The study found a strong inverse correlation between BMI and age of presentation (r = -0.28, p = 0.028), indicating that patients with greater BMI developed diverticulitis at a younger age. Most presented with Hinchey 0-1a disease (83.33%) and responded well to conservative management (90%). Surgical intervention was required in 10% of our cases, with four being urgent and two occurring during hospitalization due to persistent inflammation despite conservative treatment. During the period of the study, elective sigmoidectomy was performed in six patients (10%), mainly in patients with ≥4 episodes. Recurrence occurred in 53.3%, with a median time to recurrence of 21 months. Specifically, 26.6% of recurrences were recorded after hospitalization in our department. Recurrence was strongly associated with modifiable lifestyle factors such as smoking (OR = 3.40, p = 0.037), lack of physical activity (OR = 5.73, p = 0.002), and poor dietary adherence (OR = 0.26, p = 0.019). Higher recurrence rates were noted in patients with elevated BMI (mean: 31.1 kg/m²); however, there was no statistically significant association between recurrence and BMI or age (p = 0.13 and p = 0.40, respectively), although there was a tendency among younger and obese individuals. Patients with higher Hinchey stages had shorter recurrence intervals (χ² = 6.9, p = 0.009). Close monitoring and planned post-discharge care are essential for high-risk individuals with recurrent disease, as 40.62% did not have a follow-up colonoscopy.

Conclusions: This study highlights a growing burden of ACD among younger, overweight individuals in Greece. Conservative management is effective in most cases, but recurrence is frequent and associated with modifiable lifestyle factors. Targeted follow-up, patient education, and structured prevention strategies are essential to mitigate disease recurrence, particularly in high-risk populations.

## Introduction

Acute colonic diverticulitis (ACD), defined as inflammation of colonic diverticula, is a prevalent and increasingly encountered cause of abdominal pain in emergency departments worldwide. Its incidence has been rising steadily over recent decades, particularly among younger demographics, in both developing and industrialized countries [1]. In Western populations, diverticulosis prevalence is approximately 10% by age 40 and exceeds 50% in individuals over 60 years [2]. Of those with diverticulosis, around 7% will develop acute diverticulitis, with 1%-2% requiring hospitalization and about 0.5% necessitating surgical intervention due to complications such as abscess formation or peritonitis [1,2].

Recent epidemiological trends reveal a notable increase in hospital admissions for ACD, especially among patients younger than 60 years, while hospitalization rates for the elderly have remained relatively stable [2]. This shift challenges the traditional view of diverticulitis as a disease predominantly affecting the elderly and highlights the importance of recognizing risk factors in younger populations. The pathophysiology of diverticulosis and diverticulitis remains incompletely understood. However, several risk factors have been consistently identified, including advanced age, low dietary fiber intake, high consumption of red meat, obesity, smoking, immunosuppression, the use of nonsteroidal anti-inflammatory drugs (NSAIDs), and conditions such as inflammatory bowel disease [1-3]. Emerging evidence also correlates the complex role of gut flora in disease development and progression, suggesting potential future therapeutic targets [3]. A genetic predisposition for diverticulitis is well supported by current research, though the condition is also strongly influenced by environmental and lifestyle factors [4,5].

Computed tomography (CT) remains the gold standard for diagnosing diverticulitis and assessing disease severity. Classification of complicated diverticulitis is based on the size, localization and extent of abscesses, and the severity of peritonitis, guiding treatment decisions [1,3]. While the majority of cases follow an uncomplicated course and are managed conservatively with observation and antibiotic regimens targeting gram-negative and anaerobic bacteria, approximately 12% present with serious complications such as abscesses, strictures, fistulas, perforations, hemorrhages, or generalized peritonitis, necessitating interventions like percutaneous drainage or immediate surgery [1,3]. The recommended therapy for the perforated types with fecal or purulent peritonitis (Hinchey III and IV classification) is emergency surgery [6]. Most surgeons continue to choose Hartmann's procedure, which combines resection of the affected part of the colon with the establishment of a terminal colostomy. In terms of postoperative mortality and morbidity, several studies suggest that resection with primary anastomosis is equivalent to Hartmann's operation [7]. Recurrent diverticulitis occurs in about 20% of patients after an initial episode, although most recurrences are clinically mild. Emergency surgery is required in only a small fraction (approximately 5.5%) of patients hospitalized for recurrent disease [1]. Current clinical guidelines increasingly favor outpatient management for uncomplicated cases and recommend an individualized approach when it comes to surgical intervention based on disease severity and patient factors [2,8].

Overall, the growing incidence and healthcare burden of ACD underscore the need for continued research into its epidemiology, pathophysiology, and optimized management strategies, particularly in younger populations and those with complicated disease presentations. The primary objective of this study is to evaluate the epidemiological and clinical characteristics, management strategies, and outcomes of patients hospitalized with CT-confirmed ACD in a Greek hospital. We hypothesize that ACD increasingly affects younger and overweight individuals and that higher BMI, adverse lifestyle factors, and greater disease severity are associated with higher recurrence rates and shorter recurrence intervals. By testing this hypothesis using clinical data, this study aims to identify high-risk patient profiles and inform targeted management and follow-up strategies.

## Materials and methods

This retrospective cohort study was conducted at the Department of General Surgery, Agios Pavlos General Hospital, Thessaloniki, Greece. We included all adult patients (≥18 years) with CT-confirmed ACD admitted between January 2022 and December 2024. Patients with alternative diagnoses, including inflammatory bowel disease or colorectal malignancy, as well as those with incomplete medical records or insufficient follow-up data, were excluded. A total of 60 patients met the inclusion criteria and were included in the final analysis. A total of 60 patients met the inclusion criteria.

Patients were stratified into two age-based cohorts: Group 1 (<65 years) and Group 2 (≥65 years). The 65-year age cut-off was chosen to distinguish younger patients from the traditionally affected elderly population, as diverticulitis has historically been reported to occur predominantly after the sixth to seventh decade of life.

Data were collected using a standardized data collection form and extracted from hospital medical records. The following variables were analyzed: (a) demographic characteristics, including age, sex, body mass index (BMI), lifestyle factors (smoking status and physical activity), and comorbidities; (b) clinical presentation; (c) disease severity according to the Hinchey classification; (d) localization of diverticulitis; (e) treatment modalities (conservative, interventional, or surgical); and (f) disease recurrence. Patients were monitored for at least a year after receiving discharge instructions that included diverticular disease diet recommendations, body weight control, and advice about scheduling a colonoscopy following the remission of symptoms.

During follow-up, patients were assessed for the following parameters: (a) performance of colonoscopy, (b) adherence to lifestyle and dietary interventions, (c) disease recurrences, (d) development of complications, (e) disease-related surgical interventions, and patients' perspectives regarding their diagnosis. Recurrence was defined as a clinically confirmed episode of ACD after initial resolution, supported by imaging or hospital readmission. Patients were followed for a minimum of 12 months (median 24 months), using outpatient visits, telephone interviews, and hospital record review to capture recurrences, complications, and adherence to lifestyle modifications. Cases with incomplete follow-up or missing imaging confirmation were excluded.

Statistical analyses were performed using IBM SPSS Statistics for Windows, version 26.0 (IBM Corp., Armonk, NY). Categorical variables were compared using Chi-square or Fisher’s exact tests. Continuous variables were compared using independent-samples t-tests with Welch correction. Correlations were assessed using Pearson or Spearman coefficients. Statistical significance was set at p < 0.05.

## Results

Demographics

A total of 45% of patients were overweight (BMI: 25-30), 26.66% were Class I obese (BMI: 30-35), 6.66% were Class II obese (BMI: 35-40), and 1.66% were Class III obese (BMI > 40). Twenty percent of patients had a BMI within the normal range (18.5-25). The average BMI for the entire group of patients was 27.39 kg/m². As shown in Table 1, there was a strong inverse correlation between BMI and age at presentation (r = −0.28, p = 0.028), suggesting that individuals with greater BMI experienced diverticulitis at a younger age.

**Table 1:** Body mass index (BMI) distribution and associated demographics among patients with diverticulitis This table presents the classification of patients based on BMI categories, including the number and percentage of patients in each category, their mean BMI, and mean age. The data highlight the predominance of overweight and obesity in the study population. Notably, there is an inverse relationship between BMI and age.

BMI Category (kg/m²)	Number of Patients (n)	Percentage (%)	Mean BMI (kg/m²)	Mean Age (Years)
Normal (18.5–25)	12	20	22.27	66.75
Overweight (25–30)	27	45.00	27.39	59.50
Obese Class I (30–35)	16	26.66	31.71	56.00
Obese Class II (35–40)	4	6.66	36.82	48.75
Obese Class III (>40)	1	1.66	42.50	39.00
Total	60	100.00	—	58.23 (overall)

Comorbidities and risk factors

Comorbid conditions were reported in 75% of patients (n = 45). The most frequent were hypertension (31.66%), dyslipidemia (23.33%), and hypothyroidism (18.33%). Less common conditions included diabetes mellitus and coronary artery disease, each affecting two patients (3.3%). Gastrointestinal-related comorbidities such as irritable bowel syndrome (3.3%) and chronic constipation (6.7%) were also noted. Additionally, three patients (5%) had a history of malignancy, and two patients (3.3%) were immunosuppressed. Lifestyle factors included poor dietary habits in 58.33% of patients, smoking in 55%, alcohol consumption in 13.33%, and lack of physical exercise in 46.66%. Lifestyle-related comorbidities such as alcohol abuse (5%) were also present, highlighting the multifactorial nature of diverticulitis and the need for comprehensive management and strategies for this target group.

Clinical presentation

All patients (100%) experienced abdominal pain. Other symptoms were fever (33.33%), diarrhea (16.66%), vomiting (15%), and constipation (13.33%). The duration of symptoms before presentation was less than 24 hours in 68.33% of patients, 1-3 days in 20%, and more than three days in 11.66%. Pain was most frequently reported in the left lower quadrant (55%), followed by the hypogastric region (35%). White blood cell (WBC) counts were elevated in 65% of patients, with 50% at 11,000-14,000/mm³ and 13.33% at 15,000-19,000/mm³. Neutrophilia was observed in 63.3% of the patients. The majority of patients had increased C-reactive protein (CRP) levels, with 31.6% ranging from 0.5 to 5 mg/L and 25% between 10 and 15 mg/L.

Imaging

**Figure 1:**
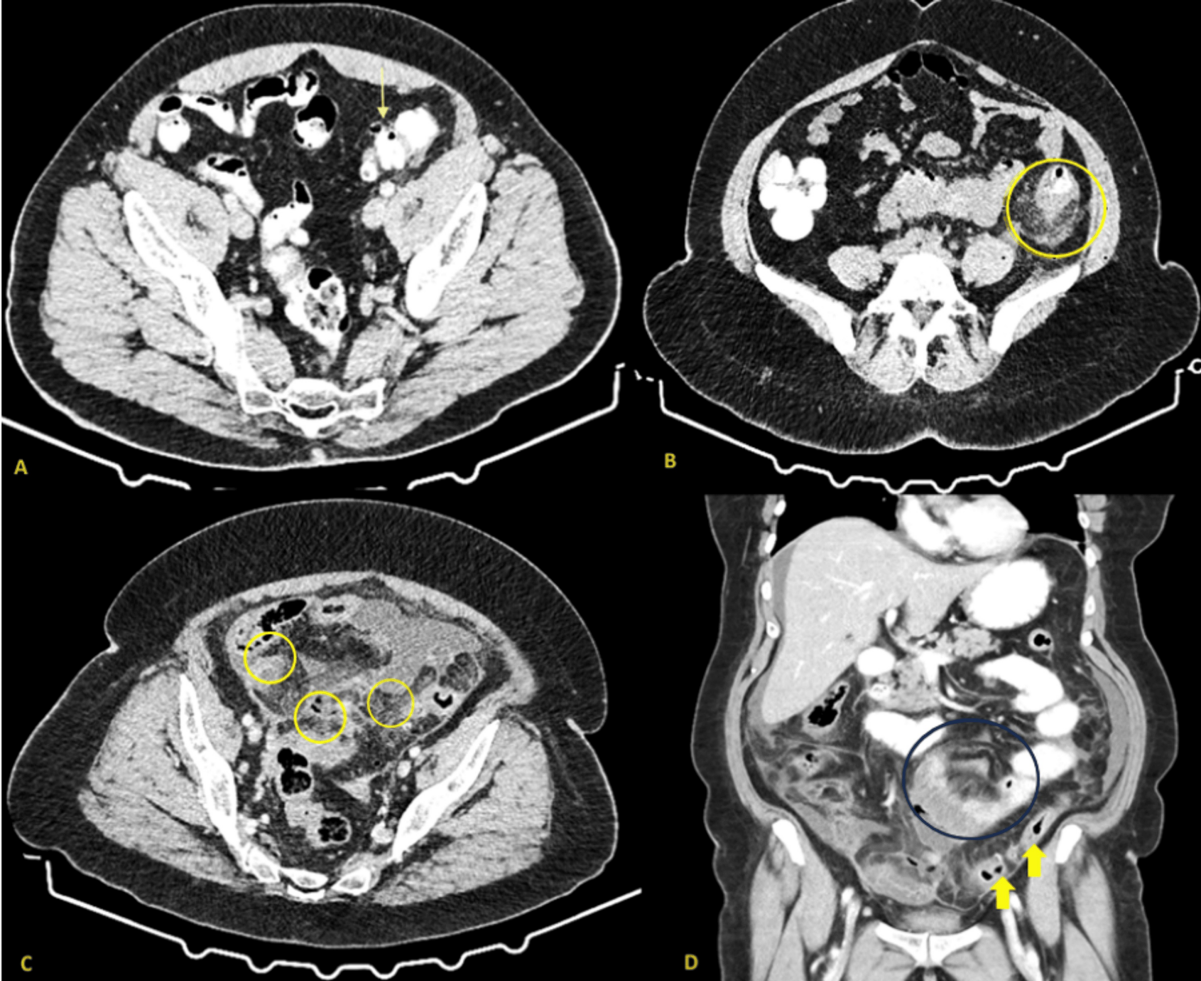
CT scans exhibiting different stages of diverticulitis (Hinchey 0-Hinchey 1b) (A) Hinchey 0 (uncomplicated diverticulitis): CT scan shows localized thickening of the sigmoid colon wall with diverticula (yellow arrow) without evidence of abscess, perforation, or pneumoperitoneum. (B) Hinchey 1a (pericolic phlegmon): CT scan demonstrates segmental colonic wall thickening of the descending colon with marked pericolic fat stranding and a small, ill-defined inflammatory phlegmon adjacent to the colon (yellow circle). There is no discrete abscess or distant fluid collection, characteristic of Hinchey 1a diverticulitis. (C (axial view), D (coronal view)) Hinchey 1b (pericolic abscess): CT scan reveals colonic wall thickening and pericolic fat stranding with well-defined, rim-enhancing pericolic abscesses measuring less than 5 cm in diameter (yellow eclipses and arrows). There was no evidence of distant abscess or generalized peritonitis, consistent with Hinchey 1b diverticulitis. Note the accumulation of small bowel dilated loops adjacent to the region of the inflammation causing paralytic ileus (black circle).

Based on results from CT, the sigmoid colon was the most common location of inflammation (55%), followed by combined sigmoid and descending colon involvement (25%). The Hinchey classification is a commonly used system for staging acute diverticulitis based on CT findings, which aids clinical care and predicts outcome. The Hinchey system, which was originally established to classify the severity of perforated diverticular illness, runs from Stage 0 (minimal clinical diverticulitis) to Stage IV (fecal peritonitis). Stage I is divided into Ia (confined pericolic inflammation or phlegmon) and Ib (pericolic or mesenteric abscess); Stage II is a distant abscess (pelvic, intra-abdominal, or retroperitoneal); Stage III is purulent peritonitis, and Stage IV is fecal peritonitis [6,9]. In this study, the majority of patients presented with Hinchey 0-1A disease (83.33%), indicating predominantly mild or localized inflammation, while only a small proportion exhibited more advanced stages requiring surgical intervention. According to the Hinchey classification, 43.33% of patients were classified as Stage 0, 40% as Stage 1a, 8.33% as Stage 1b (Figure 1), and 8.33% as Stage 2 disease, including two cases of cecal diverticulitis with abscess formation. Figure 1 depicts different CT images of patients with various Hinchey stages who participated in the study.

Localization of diverticula and diverticulitis

In our study, the sigmoid colon was involved in approximately 55% of cases, while in 25% of cases, involvement was observed at the point of transition from the descending colon to the sigmoid colon. Importantly, CT scans also revealed fewer common sites of diverticulitis, including the cecum, ascending colon, and transverse colon, each accounting for less than 5% of cases (Table 2).

**Table 2:** Anatomical localization of diverticula based on CT imaging This table outlines the anatomical distribution of diverticula and diverticulitis as identified by computed tomography. The sigmoid colon was the most commonly affected site, either in isolation or in combination with adjacent segments, consistent with typical disease patterns. (The site of the inflammation in patients with multiple sites of diverticula is noted in bold letters.)

Localization	Number of Patients (n)	Percentage (%)
Sigmoid colon	33	55.00
At the point of transition from the descending colon to the sigmoid colon	15	25.00
Descending colon	5	8.33
Cecum	2	3.33
Ascending colon	1	1.66
Ascending colon + Cecum	1	1.66
Ascending + Descending + Sigmoid colon	1	1.66
Transverse + Descending colon	1	1.66
Sigmoid + Descending colon + Cecum	1	1.66

**Figure 2:**
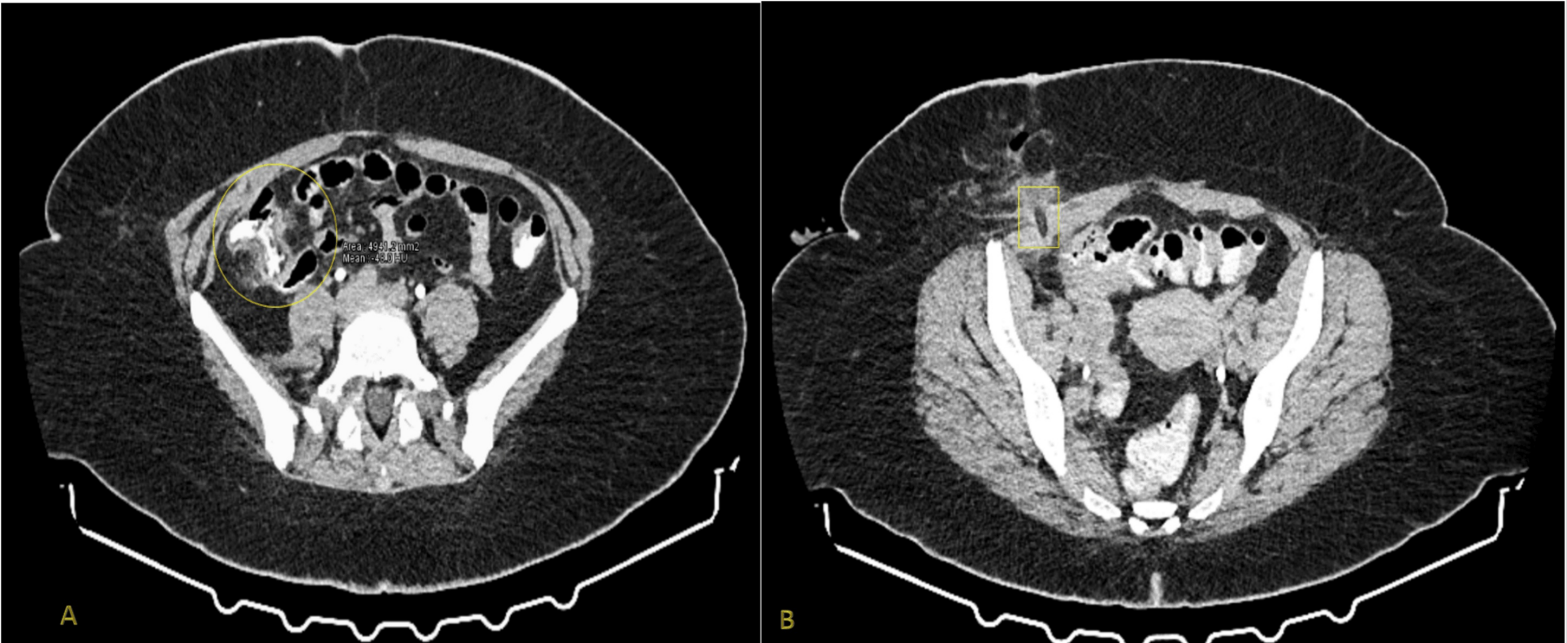
A case of Hinchey II right-sided diverticulitis and its management employing percutaneous drainage (A) Acute cecal diverticulitis with soft tissue changes of the surrounding fascia and pericolic fat stranding in the right iliac fossa (area indicated with the yellow circle). (B) The patient underwent cecostomy, in which a percutaneous drainage tube (yellow rectangle) was placed in the affected area, and an appendectomy was performed.

In our study group, isolated acute right-sided colonic diverticulitis was present in only 3.33% of our sample. In our study, we identified two cases of cecal diverticulitis with abscess formation (Hinchey II) in which surgical intervention was indicated (one of which is indicated in Figure 2).

Both cases were managed with a typical appendectomy and placement of a catheter that was inserted for percutaneous drainage of the inflamed cecal diverticulum based on the lead surgeon’s preference/experience. These patients followed a normal postoperative course and experienced no recurrence, remaining asymptomatic in our follow-up.

Treatment and outcomes

**Figure 3:**
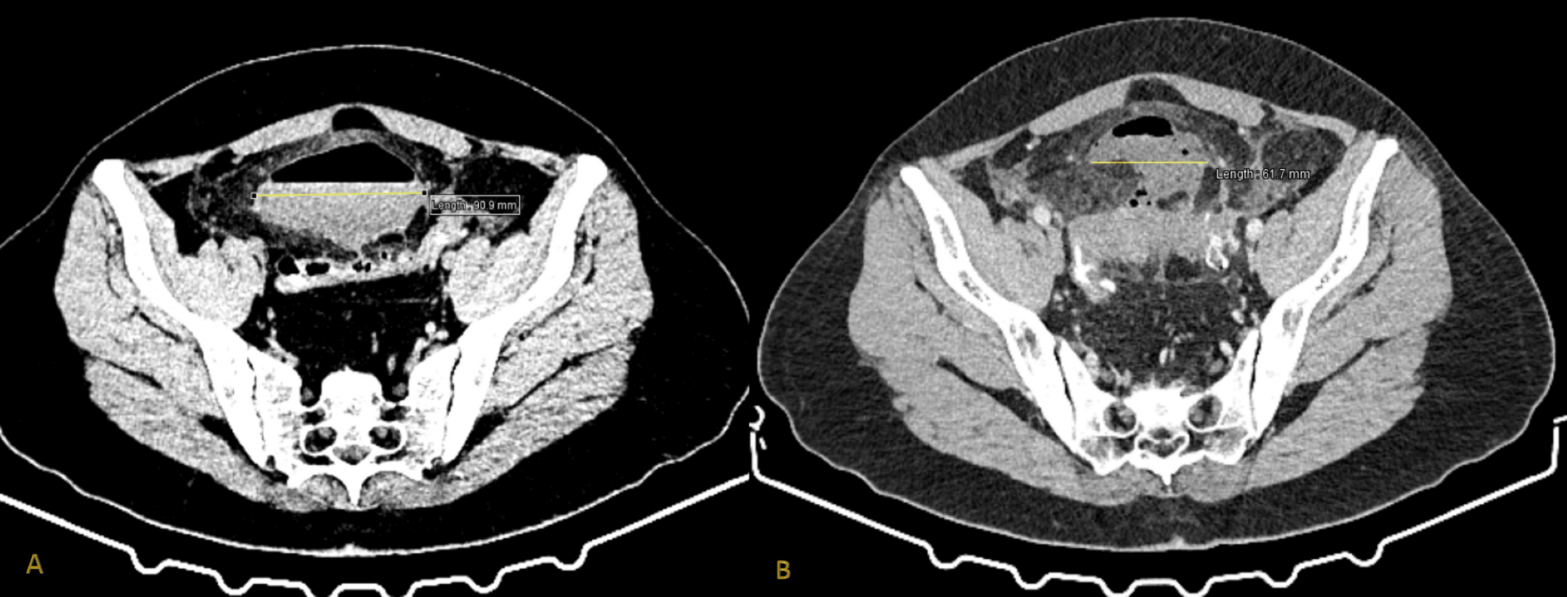
Clinical example of a Hinchey ΙΙ case and its management (A) Hinchey II acute colonic diverticulitis with pelvic abscess: Pelvic abscess measuring 9.09 cm in its maximal diameter in a young male patient who had late presentation (seven days after the onset of LLQ pain) to the emergency department. Due to the localization of the abscess, the age of the patient, and his stable condition, the patient was referred for percutaneous drainage to the gastroenterology department of another hospital, where 450 ml of purulent material was initially aspirated. (B) Post-drainage deterioration and surgical intervention: Due to the localization of the abscess, the age of the patient, and his stable condition, the patient was referred for a second percutaneous drainage of the abscess, which deteriorated to 6.17 cm in length in a new CT scan. After two incomplete drainage attempts, the patient was consequently led to the OR for Hartmann’s procedure. LLQ: Left lower quadrant.

Conservative treatment was employed in 91.67% of patients (n = 55), with intravenous antibiotics administered with intravenous (IV) antibiotics chosen according to clinical severity and suspected pathogens. In these patients, antibiotic regimens targeting gram-negative and anaerobic bacteria were administered according to disease severity. The most commonly used regimen was ciprofloxacin plus metronidazole (n = 22). Other regimens included combinations of tigecycline, tazobactam/piperacillin, and, in some cases, rifaximin was also administered during hospitalization, used mostly in patients with more severe inflammation as indicated by their CT scans and/or inflammatory markers. Patients received intravenous fluid therapy and were gradually reintroduced to oral feeding following the resolution of clinical and laboratory findings. Percutaneous drainage was performed in one case (1.66%), as seen in Figure 3.

**Figure 4:**
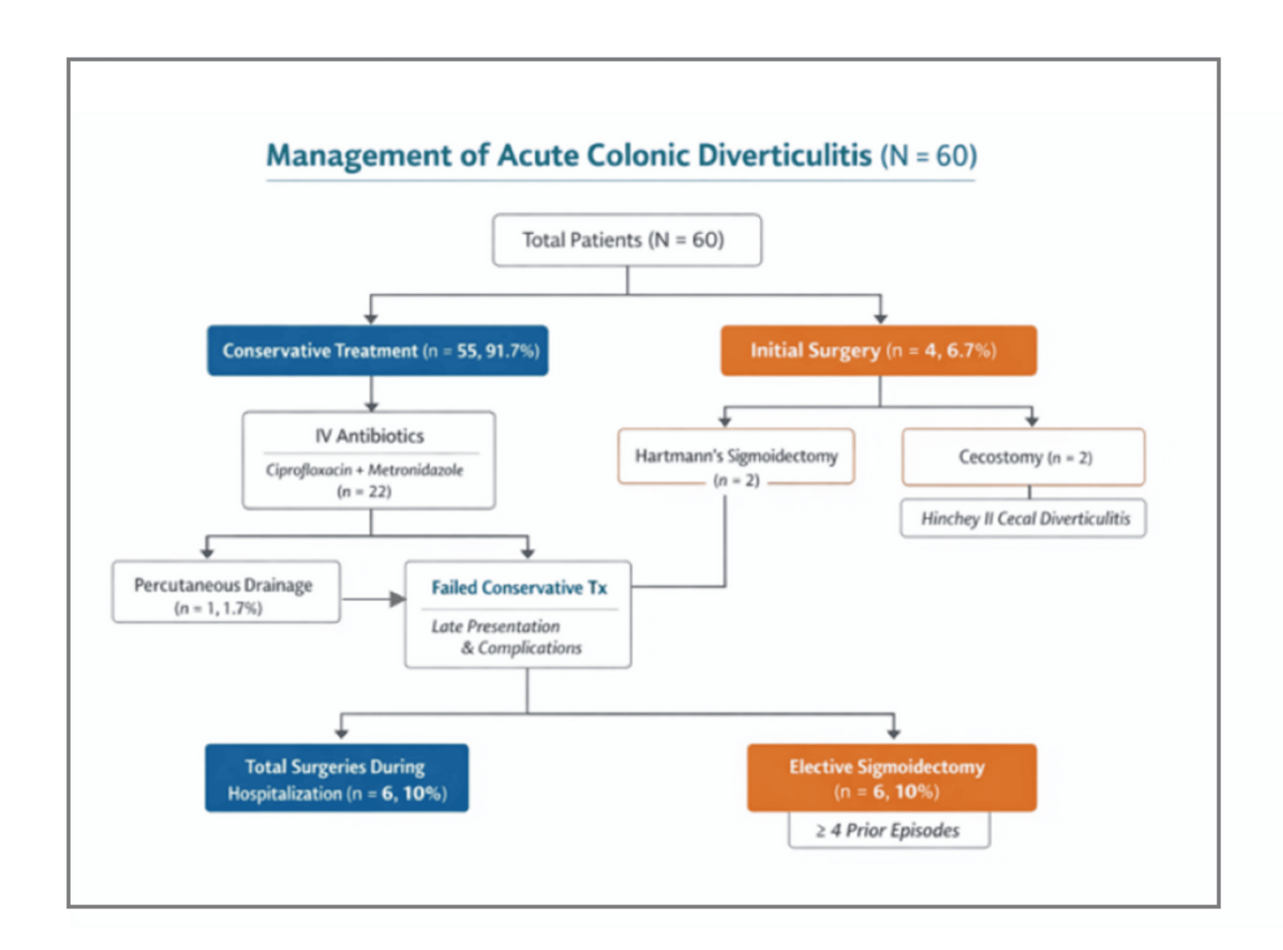
Flowchart of the management pathways of 60 acute colonic diverticulitis patients, including conservative, surgical, and elective interventions

Initial management with surgical intervention was needed in four patients (6.67%) due to the presence of large intraperitoneal pericolic abscesses on abdominal CT scan. Two of these patients underwent Hartmann's sigmoidectomy, while the other two received a cecostomy due to Hinchey II cecal diverticulitis. Out of the 60 patients, two patients presented with a higher Hinchey stage (Hinchey II) and were initially treated more conservatively, one with antibiotics (1.67%) and one with percutaneous drainage (1.67%), as explained in Figure 2. These patients required Hartmann's surgery ultimately due to complications resulting from their late presentation (six to seven days) and incomplete recession of the inflammation during their hospitalization. A total of six (10%) patients were taken to the operating room due to severe colonic diverticulitis. Over the course of three years, elective sigmoidectomy was performed in six patients (10%), most of whom had ≥4 prior episodes with relatively short intervals between diverticulitis episodes (Figure 4).

Recurrence

Recurrent diverticulitis affected more than half of the patients (n = 32, 53.3%), with a mean time to recurrence of 21 months (range: 1 month to 10 years). Moreover, 26.6% of these cases experienced recurrence post-hospitalization in our department. Recurrence of ACD was strongly associated with modifiable lifestyle factors. Smokers had significantly higher odds of recurrence compared to non-smokers (OR = 3.40, p = 0.037). Similarly, lack of physical activity was associated with markedly increased recurrence risk (OR = 5.73, p = 0.002). Interestingly, poor dietary adherence showed a lower odds ratio (OR = 0.26, p = 0.019), which may reflect limitations in dietary data collection and requires cautious interpretation. These analyses were performed using Fisher’s exact test, providing a reliable measure of association between lifestyle factors and recurrence in this cohort. There was no significant association between gender and recurrence rate (p = 0.94). Additionally, 40.62% (n = 13) of recurrent cases lacked follow-up monitoring with colonoscopy, underscoring the need for structured post-discharge care. Although colonoscopy is not suggested during the acute phase of disease, it is recommended after six to eight weeks or later, when symptoms cease, to evaluate and exclude inflammatory bowel disease or cancer, which can develop in up to 10.8% of patients with severe disease [4,10]. A higher Hinchey stage was significantly associated with more frequent recurrence (χ² = 6.9, p = 0.009) using a Chi-square test (Hinchey 0 vs. ≥1a), with more advanced stages linked to shorter recurrence intervals. Notably, Table 3 summarizes the total number of recurrences in each Hinchey stage and calculates the average interval in months between them. Patients with Hinchey stage 1a had the most recurrences, which often occurred at shorter intervals, whereas those with stage 0 had fewer and more spaced-out episodes.

**Table 3:** Summary of recurrences classified by Hinchey stage of their episode diagnosed in our clinic This table summarizes the recurrence patterns observed in patients based on Hinchey stage [6,9], including the average interval between episodes. A statistically significant association was found between Hinchey stage and recurrence, with higher stages associated with shorter recurrence intervals (χ² = 6.9, p = 0.009). Statistical test: Chi-square (2 × 2, Hinchey 0 vs. ≥ 1a).

Hinchey Stage	Number of Patients (n)	Average Recurrence Interval (Months)
0	9	~23
1a	20	~12
1b	3	~8

Immunosuppression (6.25%) and alcohol misuse (15.62%) were similarly higher in this group, albeit at lower frequencies, indicating a multifactorial etiology. To determine a potential association between BMI and disease relapse, we calculated the average age of patients based on their BMI group. Patients with recurrent diverticulitis had an inverse correlation with age at presentation (r = -0.28), indicating a trend toward earlier recurrence in patients with higher BMI. However, this association did not reach statistical significance (p = 0.13) in the Pearson test, as shown in Table 4.

**Table 4:** Relationship between body mass index (BMI) and mean age in patients with recurrent diverticulitis (N = 32) This table demonstrates the trend of earlier onset of recurrent diverticulitis in patients with higher BMI. Patients in the higher BMI categories presented with recurrence at a younger age, supporting the association between obesity and earlier, possibly more aggressive, disease recurrence among obese patients.

BMI Category (kg/m²)	Number of Patients (n)	Percentage (%)	Mean BMI (kg/m²)	Mean Age (Years)
Normal (18.5–25)	6	18.75	22.63	67.66
Overweight (25–30)	17	53.12	27.75	61.23
Obese Class I (30–35)	8	25.00	31.50	54.62
Obese Class II (35–40)	0	0.00	—	—
Obese Class III (>40)	1	3.13	42.50	39.00
Total	32	100.00	31.09 (mean)	60.00 (mean)

**Figure 5:**
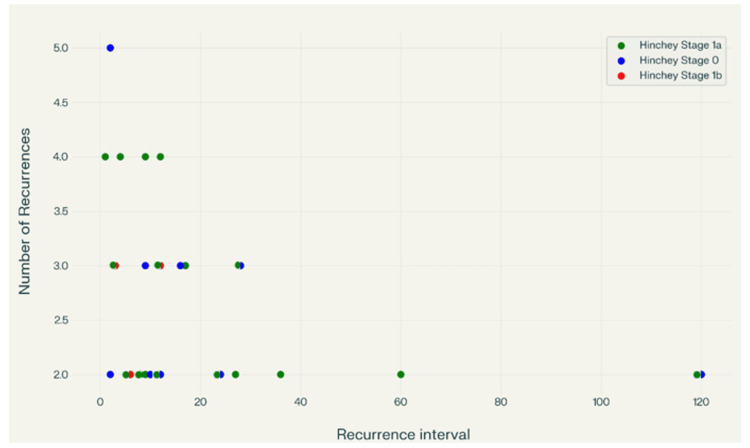
Scatter plot of the recurrent cases and their correlation to the Hinchey stage and the intervals between episodes This scatter plot visualizes the relationship between recurrence interval (in months) and the number of recurrences, grouped by Hinchey Stage (0, 1a, and 1b). This visualization suggests that recurrence patterns are highly variable across Hinchey stages, with most recurrences occurring within the first 30 months regardless of stage.

Similarly, mean age was compared, and there was no statistically significant difference between the two groups, using Fisher's test (p = 0.40). Recurrences occur over a wide range of intervals, from as early as one to two months to as late as 120 months (10 years), as exhibited in Figure 5.

Most recurrences cluster within the first 12-28 months after the initial episode. A few late recurrences (60 and 120 months) suggest long-term risk and are associated with only two episodes of diverticulitis in total. The number of recurrences per patient ranges from two to five. Higher recurrence counts (four to five) tend to occur within shorter intervals (one to four months), suggesting early frequent relapses in some patients.

**Figure 6:**
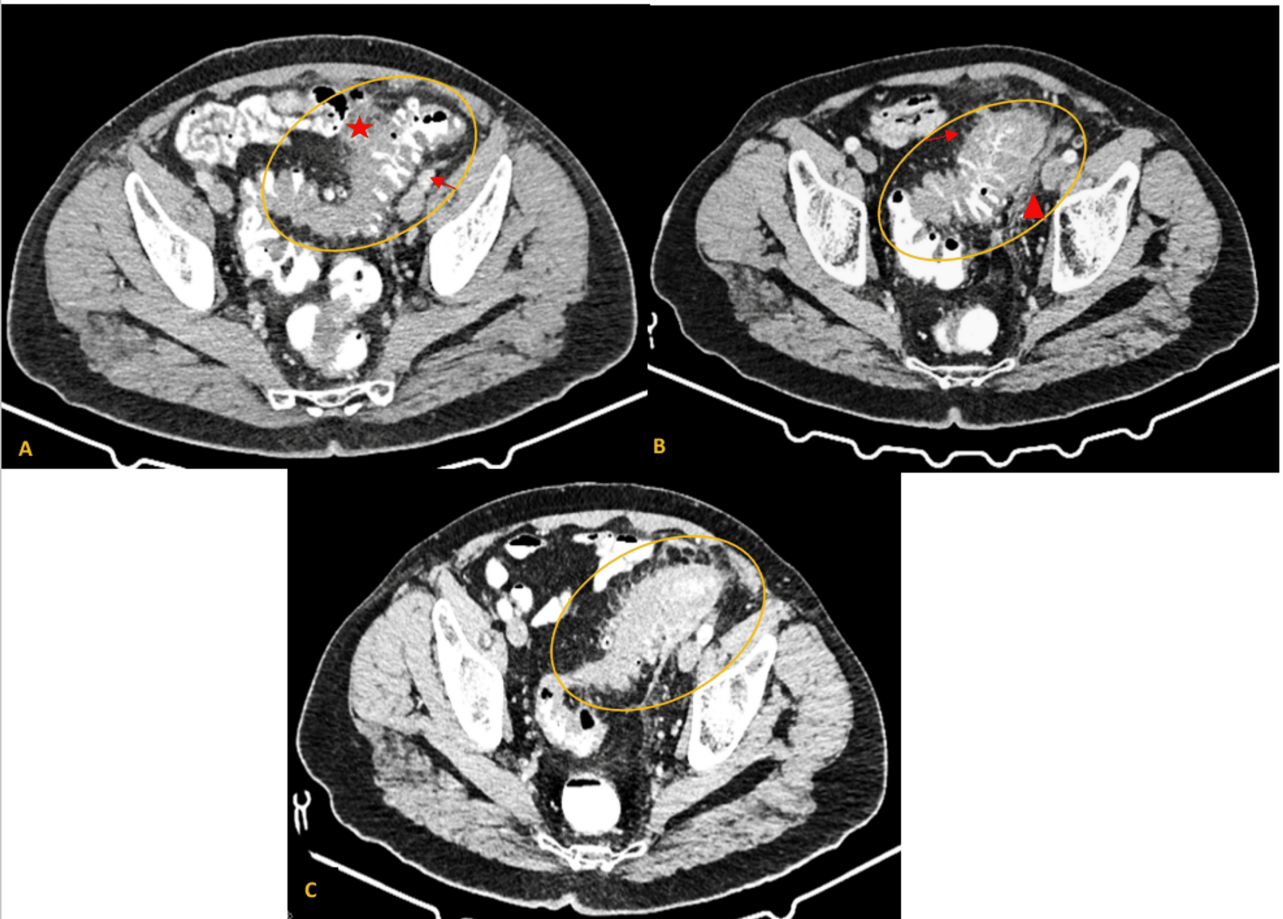
A case of 55-year-old male with two episodes of diverticulitis associated with multiple diverticula present in the sigmoid colon (Α) First episode: The sigmoid colon appears with wall multiple diverticula (yellow eclipse), wall thickening (red asterisk), and micro-fluid collections located in the left iliac fossa (red arrow) classified as Hinchey Ia. (B) Recurrent episode two years after the initial diagnosis: The middle part of the sigmoid colon appears with augmented wall thickness (yellow eclipse), multiple diverticula (red arrow), and pericolic fat stranded (red triangle) classified as Hinchey Ia. (C) Outcome after five days of conservative treatment as seen in a new CT scan: Colonic wall and pericolic fat inflammation seem to have subsided (yellow eclipse).

Considering the preceding data from the plot (Figure 5), the following observations were made. Hinchey Stage 0 (blue): The data in this group are spread across a wide range of recurrence intervals, from as short as two months to as long as 120 months. Most recurrences in Stage 0 cluster at two to three recurrences, although outliers with longer intervals (e.g., 120 months) are observed. Hinchey Stage 1a (green): Recurrence intervals for Stage 1a range from one to approximately 120 months, with a concentration of cases at shorter intervals (under 30 months). The number of recurrences in this group ranges from two to four, with a few cases reaching five. This group has the highest density of recurrences, an example of which is analyzed in Figure 6. Hinchey Stage 1b (red): Fewer data are present. Recurrence intervals are generally short (3, 6, and 12 months), and the number of recurrences ranges between two and three.

Hospitalization and annual case distribution

The mean length of hospital stay (LOS) was 5.84 days (range: 3-18), with prolonged hospitalization observed in complicated cases with higher Hinchey classification stage (Hinchey II) that consequently underwent surgery. Patients with complicated diverticulitis had a significantly longer hospital stay than those with uncomplicated disease (mean LOS 10.2 vs 5.3 days; p = 0.007). A prior history of diverticulitis was present in 50% of patients, with a mean of 2.4 prior episodes (range: 2-5). Among patients with four or more documented episodes (n = 5), two underwent elective sigmoidectomy during the study period, two were managed conservatively without surgery, and one patient with five episodes was scheduled for elective sigmoidectomy, which was performed by the end of the year. 

An analysis of the annual case distribution revealed a notable increase in the number of diverticulitis cases over the study period. In 2022, there were 11 recorded cases, followed by 16 in 2023. By 2024, the number of cases had more than doubled, reaching 33. This rising trend suggests a growing incidence of diverticulitis, particularly among younger individuals, as also reflected in the inverse relationship between BMI and age. It is important to note that in 2022, the hospital functioned partially as a designated COVID-19 facility, which likely limited the admission and diagnosis of non-COVID-related conditions, including diverticulitis. This operational constraint may partly account for the lower number of cases that year, but the sharp rise in subsequent years still points to a genuine increase in disease burden that warrants attention.

Patient perspectives

Patient-reported outcomes regarding treatment satisfaction were also assessed. Among the 60 patients, 49 (81.67%) described their treatment as effective, six (10%) considered it moderately effective, and five (8.33%) reported it as ineffective. These responses reflect a high level of perceived clinical success with the initial conservative management approach. The minority who expressed dissatisfaction or only partial benefit may correlate with those who experienced recurrence, complications, or delayed symptom resolution. Fear of recurrence was reported in 6%-10% of patients, while 3%-5% expressed fear of surgery. Dietary modification was implemented by 41.6% of patients following hospitalization. These responses underscore the importance of incorporating patient experiences into follow-up care.

## Discussion

This three-year single-center study provides a comprehensive analysis of ACD cases in a public secondary Greek hospital and highlights several key demographic, clinical, and therapeutic patterns, emphasizing age- and BMI-specific trends. The prevalence of cases in patients under 65 years (68.3%) contrasts with traditional views that diverticulitis is a disease of older age. This age shift may be influenced by lifestyle factors prevalent in younger adults, such as smoking, poor diet, and sedentary habits, all of which were overrepresented in Group 1. Comorbid conditions were present in 45 of the 60 patients, underscoring the complex clinical profiles often associated with diverticulitis. The most prevalent comorbidity was hypertension, identified in 19 patients (31.6%), followed by dyslipidemia in 14 patients (23.3%) and hypothyroidism in 11 patients.

Hypertension is linked to an increased risk of colonic diverticulosis due to vascular changes such as endothelial dysfunction and reduced blood supply, which may lead to diverticula formation. These vascular changes may weaken the colon wall at vulnerable points, facilitating the formation of diverticula [11]. Effective blood pressure control may help mitigate this risk. Some research indicates that hyperlipidemia is associated with colonic diverticulosis as part of metabolic syndrome factors like hypertension, obesity, and increased waist circumference [12]. However, the association is more consistently reported with diverticulosis rather than diverticulitis specifically. Obesity and visceral fat, which are closely linked to dyslipidemia, show a stronger and more consistent association with diverticulosis risk, possibly through proinflammatory mechanisms and chronic low-grade inflammation [12,13]. Dyslipidemia is associated with metabolic syndrome and diverticulosis; however, its specific influence on diverticulitis is not well-defined and requires further research for clarification. In our study, BMI played a central role, with over two-thirds of patients being overweight or obese. Obesity was associated with an earlier onset of diverticulitis in the overall cohort. This statistically significant inverse relationship between BMI and age supports the hypothesis that elevated BMI may accelerate disease presentation. There was also a noticeable tendency for younger patients with higher BMIs to have recurrences earlier than those with normal BMIs, although statistical significance was not achieved due to the small sample size. It is worth noting that in the non-recurrent group, a few young patients with high BMI had their first episode, so the short follow-up period could potentially have contributed to the lack of statistical significance.

Hypothyroidism has been associated with a 2.4-fold increased risk of diverticulosis, the condition of having colonic diverticula, which can become inflamed and cause diverticulitis [14]. Thyroid disorders can cause dysfunction in smooth muscle and vascular endothelium, which may affect colonic motility and contribute to diverticula formation [14,15]. One study showed that 13.5% of patients with colonic diverticula had abnormal TSH levels, indicating thyroid dysfunction, whereas hypothyroidism prevalence in the general population was about 4.6% [15]. A possible mechanism is that hypothyroidism can slow gastrointestinal motility and cause constipation, which is a known risk factor for developing diverticula in the colon [14,15]. Immunocompromised patients are at higher risk for severe or complicated diverticulitis and may present with milder symptoms, making early CT imaging essential. Corticosteroid use and other forms of immunosuppression (e.g., chemotherapy and transplant regimens) increase the risk of complications such as perforation or sepsis [4]. Emerging evidence highlights a significant genetic component in diverticular disease, including diverticulitis, with heritability estimates suggesting that 40%-53% of the risk is attributable to genetic factors, particularly evident among monozygotic twins. A family history, especially in first-degree relatives, increases the risk, suggesting both inherited and shared environmental influences. Genome-wide association studies have pinpointed various susceptibility loci, such as LAMB4 and TNFSF15, linked to processes vital for colonic wall integrity and inflammatory regulation, which are crucial in the disease's pathogenesis [4,5].

According to the literature, only history and physical examination may accurately diagnose diverticulitis in 40%-65% of cases [10]. Clinical presentations of ACD were largely consistent with classical symptomatology, with left lower quadrant (LLQ) abdominal pain being the predominant complaint in sigmoid diverticulitis. This pain was often described as sharp, cramping, or persistent, frequently accompanied by systemic signs such as fever and chills, indicating an active inflammatory or infectious process. Altered bowel habits, including diarrhea, constipation, or a combination of the two, were common and contributed to the diagnostic challenge in some cases. Other symptoms were nausea and anorexia, which were accompanied by moderate leukocytosis and indicated a systemic inflammatory response. Tenderness in the LLQ, a palpable mass, or abdominal distension may be discovered during a physical examination. In more severe cases, the abdomen exhibits rigidity, guarding, and rebound/generalized tenderness [10].

CT with intravenous contrast is necessary for confirming diagnoses, assessing disease severity, and identifying complications. While ultrasound (US) is the preferred diagnostic tool in Europe, its usage is limited in Greece and the United States. The US boasts over 90% diagnostic accuracy for detecting inflammation and other abdominal complications but has limitations, including operator dependency and potential misdiagnosis in obese patients or complex cases [10]. Current research indicates that the sigmoid colon accounts for approximately 55%-70% of diverticulitis cases in Western populations, while non-sigmoid involvement (cecum, ascending, and transverse colon) is less common (<5%-10%) and more common in some populations, such as Asians [16]. Imaging is essential for an accurate diagnosis, especially in younger patients, where right-sided diverticulitis is more common. Right-sided (non-sigmoid) diverticulitis frequently manifests as right lower quadrant pain and can mimic other acute abdominal pathologies like appendicitis or ischemic colitis [16]. This underscores the importance of thorough radiological evaluation to prevent misdiagnosis and facilitate prompt, focused treatment. It found that about 55% of cases involved the sigmoid colon, while 25% showed involvement of both the sigmoid and descending colon. The prevalence of diverticula in the sigmoid colon is attributed to its anatomical susceptibility due to higher intraluminal pressure and structural weaknesses. CT scans typically revealed segmental colonic wall thickening, pericolic fat stranding, and sometimes diverticular wall enhancement or localized abscesses. Moreover, CT imaging facilitated the detection of complications such as abscesses, fistulas, and generalized peritonitis, which necessitate more aggressive interventions, including percutaneous drainage or surgical management. The ability to stratify patients based on imaging findings has improved clinical outcomes by enabling personalized treatment plans, reducing unnecessary hospitalizations. Hinchey staging showed that most cases were uncomplicated (stages 0-1A: 83.33%), and conservative treatment was successful in the majority (54 out of 60 patients - 90%), consistent with recent literature advocating non-operative management in non-severe disease. However, in complicated cases (Hinchey II), surgical intervention occurred more frequently, likely due to comorbidity burden and delayed presentation. The Hinchey classification remains integral to the assessment and management of ACD, informing decisions regarding conservative versus surgical treatment approaches. The majority of individuals with uncomplicated diverticulitis (stages 0-Ia) can be managed conservatively. Diverticulitis with a confined abscess (stages Ib-II) can usually be treated conservatively. However, if the abscess is big or conservative treatment fails, percutaneous drainage or surgery may be necessary. For severe diverticulitis with perforation and generalized peritonitis (stages III-IV), surgery is the standard treatment [9].

Acute right-sided colonic diverticulitis presents unique management challenges, particularly when compared to left-sided disease. Isolated acute right-sided colonic diverticulitis was present in only two of our cases. The current literature consistently supports a conservative, non-operative approach as the first-line treatment for uncomplicated cases, emphasizing bowel rest and intravenous antibiotics, which have demonstrated high success rates and low recurrence - typically between 13% and 20% [17-19]. Imaging modalities, especially US and CT, play a pivotal role in distinguishing right-sided diverticulitis from acute appendicitis, thereby reducing unnecessary surgical interventions and the associated morbidity [9,20]. Most recurrences after conservative therapy remain uncomplicated and can be managed without surgery [17-19]. Surgical intervention, including right hemicolectomy or more limited resections, is generally reserved for complicated cases - such as those with abscesses, perforation, or suspicion of malignancy - or for patients experiencing frequent, severe recurrences that significantly impair quality of life [17,21].

Notably, studies indicate no significant difference in the outcomes between conservative and surgical management in terms of recurrence or hospitalization duration, further supporting a conservative-first strategy [19]. This approach reflects the more benign course of right-sided diverticulitis, with current guidelines recommending surgery only when absolutely necessary, in contrast to the more aggressive management often employed for left-sided disease [17,21]. For complicated presentations like abscesses in stable patients, percutaneous drainage combined with antibiotics is preferred, whereas in cases where perforation or hemodynamic instability occurs, immediate surgery (typically right hemicolectomy) is required [17,20]. Cecal diverticulitis often necessitates surgical resection due to its high recurrence rates and frequent misdiagnosis, as it can resemble appendicitis [17,22]. Management strategies for isolated cecal diverticulitis remain varied and controversial due to the lack of randomized trials comparing conservative and surgical interventions [22]. When cecal diverticulitis is identified during laparotomy, treatment options may include appendectomy with drainage, diverticulectomy, ileocecal resection, or right hemicolectomy, depending on the extent of inflammation and the viability of the cecum and adjacent colon [17,22]. In this study, we detected two cases of cecal diverticulitis with microabscess formation that required immediate surgery. Both patients had an appendectomy and catheter placement for percutaneous drainage, as per the lead surgeon's preference and experience. These patients had a normal postoperative course and had no recurrence, remaining asymptomatic during follow-up.

When antibiotic treatment is necessary for diverticulitis, regimens typically include broad-spectrum agents with coverage for gram-negative and anaerobic microorganisms. In the outpatient setting, mild uncomplicated diverticulitis is most commonly managed with either a combination of an oral fluoroquinolone (e.g., ciprofloxacin) and metronidazole or monotherapy with oral amoxicillin-clavulanate. The usual duration of therapy is four to seven days; however, in our practice, therapy duration is determined by initial findings at presentation and response to treatment (WBC, neutrophils, CRP, etc.) and is also tailored based on individual factors such as comorbidities, disease severity on presentation, and CT findings. Immunosuppressed patients should receive prompt antibiotic treatment with broad-spectrum agents targeting gram-negative and anaerobic bacteria, typically for 10-14 days [4]. For hospitalized patients, particularly those with complicated diverticulitis, intravenous regimens such as piperacillin-tazobactam, ampicillin-sulbactam, carbapenems, or combinations like ciprofloxacin or levofloxacin plus metronidazole are commonly used [4,10]. In our cohort of 60 patients (2022-2024), a variety of regimens were employed, including ciprofloxacin-metronidazole, tigecycline-based therapies, and piperacillin-tazobactam, with conservative treatment being effective in 90% of cases. Our approach reflects both established guidelines and individualized care strategies to optimize outcomes.

The natural history of uncomplicated diverticulitis is generally benign, with a recurrence rate ranging from 13% to 47%, supporting conservative management in many cases [23]. In patients with acute uncomplicated diverticulitis, the chance of advancing to complicated diverticulitis is 5%. Risk factors for progression consist of a baseline American Society of Anesthesiologists Physical Status Classification of III or IV, symptoms lasting over five days before presentation, the existence of vomiting, a baseline WBC count greater than 15 x 10^9^ cells/L, and CRP levels exceeding 14 mg/dL [4]. According to the bibliography, the incidence of recurrence increases with each subsequent episode [4]. It has been estimated that 8% of patients with incident disease have recurrences within one year of complete recovery, and around 20% will experience recurrences within 10 years. Following a second incident, the risk rises to 18% in one year, 40% in three years, and 55% in 10 years. In addition, it has been observed that the likelihood of recurrence is higher in patients with a history of complex diverticulitis treated effectively without surgery than in people with a history of uncomplicated diverticulitis [4].

The recurrence rate in our study was slightly elevated, estimated at around 53.3%, particularly among younger individuals (Group 1), which mirrors recent findings indicating higher recurrence in young populations. However, we lack substantial evidence to establish that age group is linked with recurrence rate in this cohort (Fisher’s test, p = 0.40). There was a negative correlation between BMI and age at presentation among patients with recurrent diverticulitis, indicating that patients with higher BMI tended to experience recurrence at a younger age. However, this association did not reach statistical significance (p-value = 0.13), likely due to the limited sample size. Modifiable factors played a significant role in recurrence. Smoking and physical inactivity were strongly associated with recurrent episodes (OR = 3.40, p = 0.037; OR = 5.73, p = 0.002, respectively), while poor dietary adherence showed a complex relationship (OR = 0.26, p = 0.019), suggesting missed opportunities for secondary prevention. Patients with a history of diverticulitis should follow a high-quality diet (high in fiber and low in red meat and sweets), maintain a normal ΒΜΙ, engage in regular physical activity, and refrain from smoking. Patients with a history of diverticulitis should also avoid using nonsteroidal anti-inflammatory drugs more than twice a week, except aspirin recommended for secondary prevention of cardiovascular disease [4].

Historically, patients with three episodes of recurrent diverticulitis were routinely offered surgical resection, with younger patients (under 40 or 50 years) facing a lower threshold. Recent guidelines now advocate for individualized decision-making regarding elective sigmoid resection, taking into account the frequency of episodes and the patient's quality of life, typically recommending surgery after multiple recurrences or complicated cases [24,25]. However, contemporary evidence shows that the highest risk of perforation occurs during the first episode (5%-25%) and decreases to less than 1% by the fifth episode [25]. Surgical outcomes favor elective sigmoid resection in selected patients with recurrent or complicated disease, with laparoscopic approaches associated with good quality of life and lower recurrence compared to conservative treatment [24]. Elective colectomy is often performed six to eight weeks following a diverticulitis episode [26]. Absolute indications for surgery in complicated diverticulitis include stenosis, fistula, and macro-abscesses that necessitate intervention, given the risk of complications. Additionally, persistent symptoms like chronic pain or bowel dysfunction that do not respond to conservative treatment warrant surgical intervention [25]. Immunosuppressed patients who have collagen-vascular diseases or chronic renal failure are prone to a higher risk of perforation and should be considered for resection after treatment of an uncomplicated episode [26]. Even after successful non-surgical treatment, chronically immunosuppressed individuals should be referred to a colorectal surgeon to consider elective resection [4]. Relative indications apply to patients with recurrent uncomplicated diverticulitis in which shared decision-making is critical, balancing the benefits of surgery against its risks and patient preferences [26]. Elective sigmoidectomy reduces the risk of recurrence to approximately 10% over two years compared to 61% with conservative management [27]. Major postoperative complications occur in 5%-10% of cases, rates comparable to conservative treatment, although patients who initially undergo conservative management and later require surgery may face higher complication risks [26].

Surgical intervention was performed in 10% of patients, primarily elective sigmoid resections for those with multiple recurrences. There is an emphasis on identifying high-risk individuals earlier to minimize recurrence and complications. Emergency surgery was required in four of 60 patients due to large abscesses, which is lower than the typical 15%-25% reported for acute diverticulitis necessitating urgent intervention [28]. Current guidelines indicate that while many diverticular abscesses can be managed conservatively with antibiotics and percutaneous drainage, emergency surgery remains indicated in cases of large abscesses, generalized peritonitis, or clinical deterioration [29]. In international practice, Hartmann’s procedure remains a common option for emergency resection, particularly in the setting of severe infection or hemodynamic instability, although primary anastomosis is increasingly used in selected cases [7,30]. A three-year follow-up randomized controlled trial indicates that in hemodynamically stable, immunocompetent patients, primary anastomosis is superior to Hartmann's procedure as treatment for perforated diverticulitis with respect to long-term stoma-free rate, parastomal hernias, and hospitalization [31].

Strengths and limitations

Strengths of this study include comprehensive data collection and a three-year follow-up of a well-characterized cohort. This study is limited by its retrospective design and relatively small sample size, which may affect generalizability. The single-center nature of the study reflects local patient demographics, referral patterns, and institutional clinical practices, which may differ from those of other regions or healthcare systems, potentially limiting external validity. Additionally, variations in antibiotic protocols and follow-up strategies reflect the absence of standardized management protocols. Future prospective studies are warranted to assess the efficacy of standardized post-discharge protocols.

## Conclusions

This study demonstrates that ACD is no longer confined to elderly populations, with the majority of cases in our series occurring in patients under 65 years of age. Obesity is a major modifiable factor associated with younger age at onset, recurrent disease, and poor outcomes. Conservative treatment remains highly effective, even in older and comorbid patients, provided that early diagnosis and supportive care are offered, while surgery is favored in those with complicated disease or significantly impaired quality of life. Recurrent episodes appear to be linked to poor lifestyle choices and failure to comply with medical recommendations. Relapses are common and, to some extent, often predictable based on behavior patterns and monitoring patterns. Lifestyle counseling following discharge, dietary intervention, and routine follow-up (including colonoscopy) should be integral to diverticulitis management, especially for patients at risk of recurrence. Simple lifestyle interventions and close monitoring can reduce recurrence rates, readmissions, and morbidity in individuals with acute diverticulitis. Public health strategies targeting obesity, sedentary lifestyle, and smoking may help reduce the burden of this increasingly common disease.
